# Thrombin Cleavage of *Plasmodium falciparum* Erythrocyte Membrane Protein 1 Inhibits Cytoadherence

**DOI:** 10.1128/mBio.01120-16

**Published:** 2016-09-13

**Authors:** Mark R. Gillrie, Bernard Renaux, Eleanor Russell-Goldman, Marion Avril, Andrew J. Brazier, Koichiro Mihara, Enrico Di Cera, Danny A. Milner, Morley D. Hollenberg, Joseph D. Smith, May Ho

**Affiliations:** a Department of Microbiology, Immunology and Infectious Diseases, University of Calgary, Calgary, Alberta, Canada; b Department of Physiology and Pharmacology and Department of Medicine, University of Calgary, Calgary, Alberta, Canada; c Department of Pathology, Harvard Medical School, Boston, Massachusetts, USA; d Center for Infectious Disease Research, Seattle, Washington, USA; e Department of Biochemistry and Molecular Biology, Saint Louis University School of Medicine, St. Louis, Missouri, USA

## Abstract

*Plasmodium falciparum* malaria remains one of the most deadly infections worldwide. The pathogenesis of the infection results from the sequestration of infected erythrocytes (IRBC) in vital organs, including the brain, with resulting impairment of blood flow, hypoxia, and lactic acidosis. Sequestration occurs through the adhesion of IRBC to host receptors on microvascular endothelium by *Plasmodium falciparum* erythrocyte membrane protein 1 (PfEMP1), a large family of variant surface antigens, each with up to seven extracellular domains that can bind to multiple host receptors. Consequently, antiadhesive therapies directed at single endothelial adhesion molecules may not be effective. In this study, we demonstrated that the serine protease thrombin, which is pivotal in the activation of the coagulation cascade, cleaved the major parasite adhesin on the surface of IRBC. As a result, adhesion under flow was dramatically reduced, and already adherent IRBC were detached. Thrombin cleavage sites were mapped to the Duffy binding-like δ1 (DBLδ1) domain and interdomains 1 and 2 in the PfEMP1 of the parasite line IT4var19. Furthermore, we observed an inverse correlation between the presence of thrombin and IRBC in cerebral malaria autopsies of children. We investigated a modified (R67A) thrombin and thrombin inhibitor, hirugen, both of which inhibit the binding of substrates to exosite I, thereby reducing its proinflammatory properties. Both approaches reduced the barrier dysfunction induced by thrombin without affecting its proteolytic activity on PfEMP1, raising the possibility that thrombin cleavage of variant PfEMP1 may be exploited as a broadly inhibitory antiadhesive therapy.

## IMPORTANCE

*Plasmodium falciparum* malaria is the third leading cause of mortality due to a pathogen, with 214 million people infected and 438,000 deaths annually. The adhesion of *Plasmodium falciparum*-infected erythrocytes (IRBC) to microvascular endothelium is a major pathological process in severe malaria. While the recent implementation of artemisinin-based antimalarial therapy for severe malaria improves patient survival by targeting all parasite stages, antiparasite drugs alone may not immediately reverse pathophysiological processes in occluded vessels. Here we show that thrombin, an enzyme intimately involved in the clotting process, cleaves the main parasite adhesin expressed on the surface of IRBC, thereby preventing and reversing the binding of IRBC to endothelial cells. This beneficial effect of thrombin can be achieved by modified thrombins that cause significantly less clotting and vessel leakage while preserving the ability to cleave the parasite protein. Our results provide the basis for using modified thrombins as adjunctive therapy in severe malaria.

## INTRODUCTION

In the past 15 years, mortality due to the protozoan parasite *Plasmodium falciparum* has decreased by 60% globally (1), and this is largely attributed to the efficacy of antimalarial combination therapy that includes artemisinin derivatives (2, 3). The drug has a rapid parasiticidal action and targets all stages of the erythrocytic cycle of the parasite. Parasite clearance is usually achieved within the first 24 to 36 h after hospital admission, during which most of the deaths occur. Unfortunately, although not entirely unexpectedly, artemisinin-resistant parasites have now emerged in Southeast Asia, the hotbed of antimalarial resistance (4). Moreover, resistance has spread to some of the drugs that are used in combination with artemisinins—e.g., piperaquine (5). There is therefore an urgent need to develop an adjunctive therapy that is directed toward major pathogenic processes rather than focusing solely on the elimination of parasites.

One target for adjunctive therapy is the sequestration of *P. falciparum*-infected erythrocytes (IRBC) in the microcirculation of vital organs, leading to impairment of blood flow, hypoxia, and lactic acidosis (6). Sequestration results from the adhesion, or cytoadherence, of IRBC to vascular endothelial cells, and the process is mediated by the variant parasite ligand *Plasmodium falciparum* erythrocyte membrane protein 1 (PfEMP1) and endothelial receptors, of which a number have been implicated in severe disease (7, 8). Evidence for cytoadherence as a major pathological process comes not only from detailed histopathological studies of human postmortem tissues (9, 10) but also from imaging the microcirculation in infected patients (11), as well as clinical studies showing decreased cerebral perfusion (12) and lactate production (13) in patients with severe falciparum malaria. The importance of cytoadherence is supported further by the increased prevalence of protective point mutations in the hemoglobin gene within human populations living in areas where malaria is endemic: e.g., hemoglobin C and hemoglobin AS. The protection is due at least in part to an abnormal display of PfEMP1 on the surface of IRBC, which profoundly affects their ability to adhere to endothelial cells (14, 15). These observations clearly indicate that reducing cytoadherence is a therapeutic option for improving clinical outcomes.

In pediatric patients with cerebral malaria (CM), coagulopathy leading to fibrin deposition may also be a contributing factor to disease severity (16). Specifically, adherent IRBC have been shown to induce tissue factor (TF) production by endothelial cells *in vitro*, thus initiating the extrinsic coagulation pathway (17). Activation of the intrinsic pathway is also found in adult patients with severe malaria (18) and can be initiated *in vitro* by the release of the contents of the parasite digestive vacuole (19). Moreover, the secreted parasite product *Plasmodium falciparum* histidine-rich protein (PfHRP-2) contributes to a procoagulant environment by inhibiting the activity of antithrombin III (20), and the endothelial protein C receptor (EPCR)-binding cysteine-rich interdomain region α1.4 (CIDRα1.4) domain of PfEMP1 inhibits the generation of activated protein C (APC) (21, 22), a key anticoagulant protein. These *in vitro* observations and the demonstration of fibrin deposition in >85% of cerebral microvessels in children who died from cerebral malaria (16) make a strong case for the potential pathological significance of microvascular thrombosis in pediatric cerebral malaria.

Central to the interplay between the malaria parasite and the human coagulation system is the serine protease thrombin, which directly coordinates the balance between a procoagulant state by fibrin generation and an anticoagulant state by the activation of protein C. Additionally, depending on the site of substrate cleavage, thrombin can induce either cytoprotection of endothelial barrier function or proinflammatory effects and barrier dysfunction through activation of protease-activated receptor-1 (PAR1), -3, and -4 signaling (23). Recognition of substrates such as fibrinogen and PAR1 requires extensive interactions with the active site and exosite I on thrombin (24).

In previous experiments with the parasite lines IT4var19 and IT4var07 (21), we observed that the exposure of endothelial cell-parasite cocultures to thrombin led to the detachment of IRBC from primary human brain and lung microvascular endothelial cells (M. R. Gillrie et al., unpublished data). The observation suggested a previously unappreciated proteolytic activity of thrombin on either endothelial receptors or PfEMP1. In this study, we present functional and biochemical findings to demonstrate cleavage of multiple PfEMP1 variants by thrombin. Engineered thrombins with minimal PAR1 activation but preservation of the proteolytic activity against PfEMP1 may serve as a potential adjunctive approach for severe malaria.

## RESULTS

### Thrombin inhibits IRBC adhesion to microvascular endothelial cells under shear stress.

**FIG 1:**
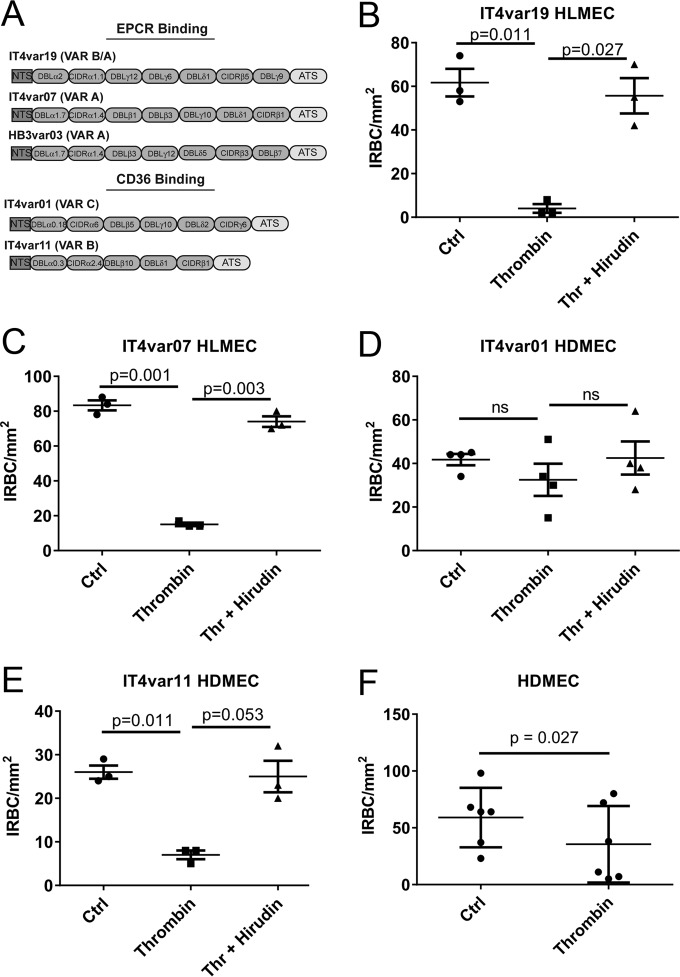
Thrombin inhibits adhesion of *P. falciparum* IRBC with diverse binding phenotypes to microvascular endothelial cells. (A) Schematic of the extracellular domain architecture for the 5 laboratory-adapted *P. falciparum* parasite lines used in this study. EPCR and CD36 binding parasite lines expressing a predominant *var* gene were derived from the parental parasite lines FCR3/IT4 and HB3. Each *var* gene is composed of a cysteine-rich interdomain region (CIDR) and Duffy-binding like (DBL) domains. (B to E) Adhesion of IRBC from IT4-derived parasite lines pretreated with 10 nM thrombin with or without 100 nM hirudin for 30 min at 37°C. Adhesion assays on HLMEC or HDMEC were performed using IRBC at 1% hematocrit and 4 to 5% parasitemia in a parallel-plate flow chamber at 1 dyne/cm^2^ (*n* = 3, except for panel E, where *n* = 4). Ctrl, control. Results are shown as mean ± SEM and were analyzed by ANOVA followed by *post hoc* multiple comparisons using Tukey’s test. (F) Adhesion of six cryopreserved clinical parasite isolates obtained from infected adult Thai patients on HDMEC. IRBC were pretreated with 25 nM thrombin before being used in the flow chamber assay. Results are shown as mean ± SEM and were analyzed by Student’s paired *t* test.

To investigate if thrombin has any intrinsic effect on IRBC, the adhesion of diverse *P. falciparum* binding phenotypes to endothelial cells was studied in a parallel-plate flow chamber using four previously characterized IT4 laboratory-adapted parasite lines that express a single predominant PfEMP1 (25, 26) (Fig. 1A). The parasite lines IT4var19 and IT4var07, which adhere to EPCR, were tested on primary human lung microvascular endothelial cells (HLMEC) (21), while the parasite lines IT4var01 and IT4var11 and clinical parasite isolates that preferentially bind to CD36 (27) were tested on primary human dermal microvascular endothelial cells (HDMEC). IRBC were preincubated with 10 nM thrombin with or without the highly specific thrombin inhibitor hirudin (100 nM) for 30 min at 37°C before being infused over an endothelial monolayer at 1% hematocrit and a shear stress of 1 dyne/cm^2^. The dose of thrombin (10 nM) used in these experiments was well within the levels detected in the circulation during fibrinogenesis in humans (200 to 300 nM) (28). As shown in Fig. 1B to E, thrombin inhibited the binding of IT4var19, IT4var07, and IT4var11 but not IT4var01 (*n* = 3 to 4 for each parasite line). The proteolytic effect was completely reversed in the presence of hirudin. More importantly, thrombin also inhibited the adhesion of 4 of 6 randomly selected clinical parasite isolates from adult Thai patients to HDMEC (Fig. 1F). The percentages of inhibition for the 4 responders were 41, 78, 81, and 83%, and those for the 2 nonresponders were 0 and 18%.

### Thrombin cleaves PfEMP1 from the surface of *P. falciparum* IRBC.

**FIG 2:**
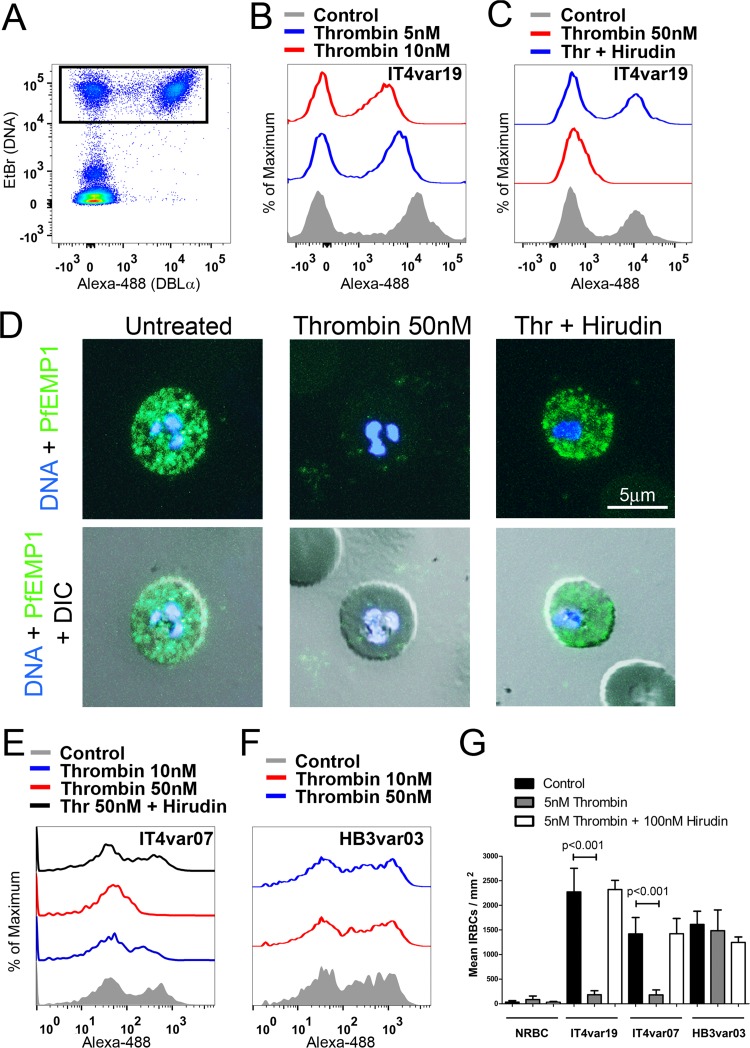
Thrombin cleaves IT4var19 PfEMP1. (A) Flow cytometric analysis of PfEMP1 expression on IRBC from IT4var19 at the trophozoite stage by dual labeling with ethidium bromide (EtBr) and a rabbit anti-DBLα2 polyclonal antibody followed by Alexa-488-goat anti-rabbit MAb. About 50 to 60% of IRBC from IT4var19 express the variant-specific DBLα2 (upper-right-hand quadrant). (B and C) Flow cytometric analysis of PfEMP1 expression of IRBC treated with (B) 5 and 10 nM thrombin or (C) 50 nM thrombin (Thr) with or without 100 nM hirudin for 30 min at 37°C. (D) Immunofluorescence microscopy of PfEMP1 expression on live IRBC from panel C, showing DBLα2 (green) in relation to DNA (blue) and malaria pigment (black in differential inference contrast [DIC] images). Images were taken at ×600 magnification. (E and F) Flow cytometric analysis of PfEMP1 expression on IRBC from (E) IT4var07 and (F) HB3var03. Results shown are representative of 2 experiments for panels A to F. (G) Thrombin inhibits the adhesion of IRBC from IT4var19 and IT4var07 but not HB3var03 on HBMEC in a static binding assay (*n* = 3). NRBC, normal red blood cells. Results are shown as mean ± SEM and were analyzed by ANOVA followed by *post hoc* multiple comparisons using Tukey’s test.

The inhibition of IRBC binding under shear stress by thrombin, a serine protease, suggests the IRBC adhesin PfEMP1 might be proteolytically cleaved by thrombin. The cleavage of PfEMP1 by 5, 10, and 50 nM thrombin was demonstrated by flow cytometry using a polyclonal antibody that targets the Duffy binding-like α2 (DBLα2) domain of IT4var19 (Fig. 2A to C). While PfEMP1 cleavage was 100% at 50 nM thrombin, there were consistent drops in mean fluorescent intensity (MFI) of 50 and 75% for PfEMP1 expression at 5 and 10 nM thrombin, respectively. Thrombin did not induce any morphological changes in IRBC as examined by confocal fluorescence microscopy, but consistent with the flow cytometry results, there was a loss of punctate staining by anti-DBLα2 (Fig. 2D). Similarly, cleavage of surface PfEMP1 of the parasite line IT4var07 was demonstrated by flow cytometry using a *var*-specific polyclonal antiserum (Fig. 2E). In contrast, no cleavage of the PfEMP1 of the parasite line HB3var03 (Fig. 1A) by thrombin was seen (Fig. 2F), consistent with the lack of effect of thrombin on the adhesion of this parasite line to an immortalized brain microvascular endothelial cell line (THBMEC) in a static binding assay (29) (Fig. 2G). This parasite line was tested in a static binding assay as it did not adhere well to HLMEC under flow conditions (<10 adherent IRBC/mm^2^). These results demonstrate that not only does thrombin functionally impair IRBC adhesion to endothelial cells, it likely does so by directly cleaving the primary parasite adhesin PfEMP1.

### Thrombin cleaves PfEMP1 of IT4var19 at DBLδ1 and interdomains 1 and 2.

**FIG 3:**
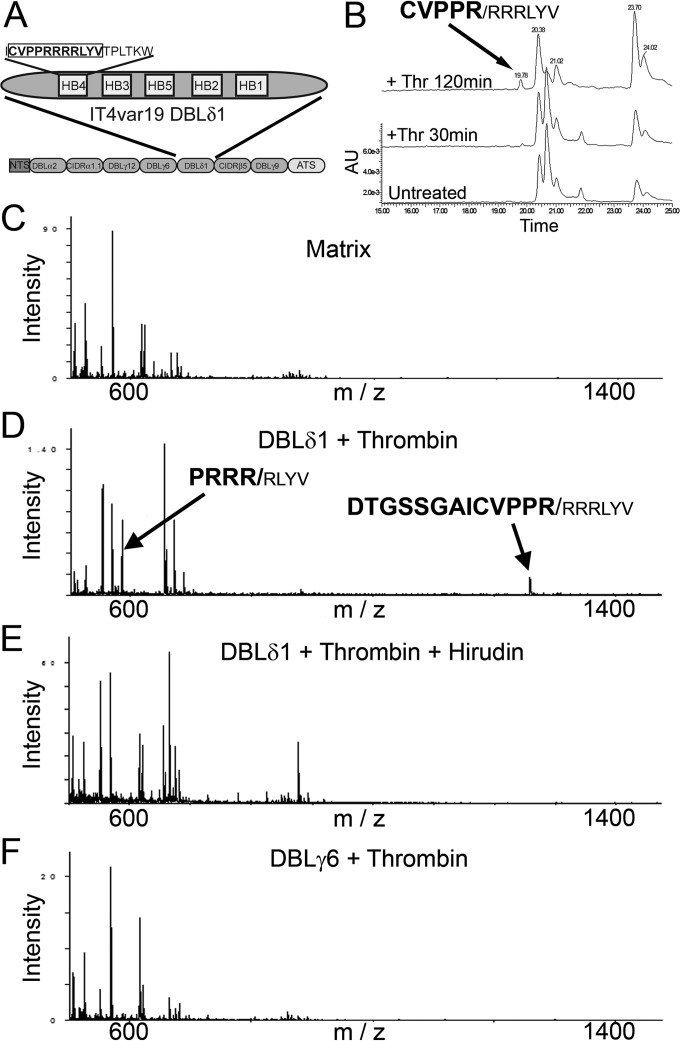
Thrombin cleaves DBLδ1 domain of IT4var19. (A) Schematic of the DBLδ1 domain of IT4var19 showing the predicted cleavage sequence CVPPRRRRLYV. **(**B) HPLC analysis of a synthetic peptide from DBLδ1 (aa 1768 to 1778) treated with 25 nM thrombin for 30 min and 2 h. The molecular mass (±0.01 Da) of each of the resulting fragments, as determined by tandem time of flight mass spectrometry (TOF/TOF MS), is indicated above each peak (*n* = 2). (C) TOF/TOF MS analysis of the MS α-cyano-4-hydroxycinnamic acid matrix alone. (D) TOF/TOF MS analysis of full-length recombinant IT4var19 DBLδ1 (5 µg) treated with thrombin (250 nM for 2 h at 37°C) demonstrating cleavage products at 584.36 and 1,259.60 Da (±0.01 Da; *n* = 2). Cleavage products are indicated by arrows with corresponding amino acid sequences displayed above. (E) No cleavage products were found when thrombin was preincubated with hirudin (1 µM for 10 min) prior to incubation with the DBLδ1 domain (*n* = 2)**.** (F) TOF/TOF MS analysis of full-length recombinant IT4var19 DBLγ6 demonstrating no cleavage fragments in the presence of thrombin (250 nM for 2 h at 37°C; *n* = 2).

To determine potential thrombin cleavage sites within PfEMP1 proteins, we performed a detailed search of 30 published *var* gene sequences with partially defined binding phenotypes using the algorithms Prosper (30) and ExPasy Peptide Cutter (31), which are highly specific (>95%) but not sensitive (<70%) for thrombin cleavage sites. Neither algorithm revealed a potential thrombin cut site in IT4var19 PfEMP1, but they did demonstrate 34 potential cut sites in 14 of the remaining 29 *var* genes (Table 1). We also performed more sensitive but less-specific manual searches of the protein sequences for proline-arginine (PR) and more specifically the PRXXR sequence that has been reported as a preferred site for thrombin activity (32). This search revealed a large number of PR sequences both within particular PfEMP1 DBL and CIDR domains as well as in the interdomain regions. However, the only potential thrombin cleavage site fulfilling the PRXXR sequence requirements of IT4var19 was a relatively conserved PRRRR sequence in the DBLδ1 domain (Fig. 3A). Interestingly, for the five parasite lines tested in this study, the presence of the PRRRR sequence in the DBLδ1 domain predicted the ability of thrombin to inhibit adhesion (Table 1). Thus, thrombin did not affect adhesion of the two parasite lines that do not express the thrombin-susceptible DBLδ1 sequence (IT4var01 and HB3var03).

**TABLE 1:** Analysis of *P. falciparum var* genes with partially defined binding phenotypes for the presence of thrombin cleavage sites

*var* gene type^a^	Genome	Geographic origin	Binding phenotype		No. of potential thrombin cleavage sites by^b^:		No. of potential thrombin cleavage sites with sequence^b^:		Presence of^c^:			Thrombin cleavage
			CIDR1	Other	Prosper	Peptide Cutter	PR	PRXXR	DBLδ1	KPRGPA	GLGRSL	
IT4var06 (DC8)	IT4/FCR3	Southeast Asia	EPCR	?	0	11	7	1^d^	Yes	No	No	ND^e^
IT4var19 (DC8)	IT4/FCR3	Southeast Asia	EPCR	?	0	0	7	1^d^	Yes	Yes	Yes	Yes
IT4var20 (DC8)	IT4/FCR3	Southeast Asia	EPCR	?	0	1	7	0	Yes	No	No	ND
IT4var32b (DC8)	IT4/FCR3	Southeast Asia	EPCR	?	0	0	7	1^d^	Yes	Yes	Yes	ND
DD2var47 (DC8)	DD2	Southeast Asia	EPCR	?	0	1	6	0	Yes	No	No	ND
IGHvar19 (DC8)	IGH	India	EPCR	?	0	0	6	1^d^	Yes	No	No	ND
RAJ116var08 (DC8)	RAJ116	India	EPCR	?	0	0	4	0	No	No	No	ND
PFCLINvar30 (DC8)	PFCLIN	Africa	EPCR	?	0	0	4	0	No	No	No	ND
PFD0020c (DC8)	3D7	Unknown	EPCR	?	0	2	10	2 (1^d^)	Yes	No	No	ND
PF08_0140 (DC8)	3D7	Unknown	EPCR	?	1	1	7	0	Yes	No	No	ND
RAJ116var11 (DC8)	RAJ116	India	EPCR	?	0	0	6	1^d^	Yes	No	No	ND
PFCLINvar31 (DC8)	PFCLIN	Africa	EPCR	?	2	1	6	1	No	No	No	ND
MAL6P1.316 (DC8)	3D7	Unknown	EPCR	?	0	1	6	0	No	No	No	ND
IT4var01	IT4/FCR3	Southeast Asia	CD36	ICAM-1	0	1	3	0	No	No	No	No
IT4var11	IT4/FCR3	Southeast Asia	CD36	?	0	0	3	1^d^	Yes	No	No	Yes
IT4var14	IT4/FCR3	Southeast Asia	CD36	ICAM-1	0	0	7	1^d^	Yes	No	No	ND
IT4var16	IT4/FCR3	Southeast Asia	CD36	ICAM-1	1	2	4	1^d^	Yes	No	No	ND
IT4var31	IT4/FCR3	Southeast Asia	CD36	ICAM-1	0	0	3	1	No	No	No	ND
IT4var33	IT4/FCR3	Southeast Asia	CD36	?	1	1	2	1^d^	Yes	No	No	ND
IT4var39	IT4/FCR3	Southeast Asia	CD36	?	0	0	3	1^d^	Yes	No	No	ND
IT4var44	IT4/FCR3	Southeast Asia	CD36	?	0	0	4	1^d^	Yes	No	No	ND
IT4var07	IT4/FCR3	Southeast Asia	EPCR	ICAM-1	0	3	3	1^d^	Yes	No	Yes	Yes
HB3var03	HB3	Central America	EPCR	ICAM-1	0	0	5	1	No	No	No	No
PFD1235wD4	3D7	Unknown	EPCR	ICAM-1	1	1	8	2 (1^d^)	Yes	No	No	ND
IT4var02	IT4/FCR3	Southeast Asia	?	PECAM-1	0	0	8	1	No	No	No	ND
PF11_0008	3D7	Unknown	?	PECAM-1	0	0	8	1	No	No	No	ND
PF13_0003	3D7	Unknown	?	Rosette	1	0	10	1	No	No	No	ND
HB3var06	HB3	Central America	?	Rosette	0	0	6	0	No	No	No	ND
IT4var09	IT4/FCR3	Southeast Asia	EPCR	Rosette	0	0	4	1^d^	Yes	No	No	ND
IT4var60	IT4/FCR3	Southeast Asia	?	Rosette	1	0	6	0	No	No	No	ND
Total (*n* = 30)					8	26	170	23	18	2	3	3/5

a Thirty *var* genes encoding unique PfEMP1 were selected for comparison based on (i) being closely related to IT4var19 (domain cassette 8 [DC8] *var* genes—i.e., *var* genes with DC8 [NTS-DBLα2-CIDRα1-DBLβ12-DBLγ4/6]), (ii) having partially defined binding phenotypes, and (iii) having available sequence data that were previously catalogued.

b The Prosper and Peptide Cutter algorithms or manual searches of PR sequences (PR or PRXXR) were performed to identify potential thrombin cleavage sites.

c The presence of DBLδ1 containing PRRRR or interdomain KPRGPA and GLGRSL sequences was also determined.

d PRRRR sequence within a DBLδ1 domain.

e ND, not determined.

The presence of the PRRRR sequence within the highly conserved homology box 4 and frequently found DBLδ1 domain suggests that this candidate cleavage site is likely common to a large number of *var* genes (33). To investigate whether the PRRRR sequence of DBLδ1 of IT4var19 was targeted by thrombin for cleavage, we synthesized the peptide CVPPRRRRLYV (amino acids [aa] 1768 to 1778) and subjected it to high-performance liquid chromatography/matrix-assisted laser desorption ionization (HPLC/MALDI) analysis after exposure to thrombin. The results showed that thrombin cleaved the synthetic peptide after arginine 1772 (CVPPR/RRRLY), confirming that the PRRRR sequence is a possible target of the proteolytic activity of thrombin (Fig. 3B). To confirm cleavage of DBLδ1, we also subjected the entire recombinant IT4var19 DBLδ1 domain to thrombin cleavage followed by matrix-assisted laser desorption (MALDI) identification of the resulting proteolytic fragments. Exposure of full-length DBLδ1 to thrombin demonstrated a minor cleavage at arginine 1772 (PPR/RRR) and a major cleavage site at 1774 (PPRRR/R) (Fig. 3C), both of which cleavage products were not found when thrombin was inhibited by hirudin (Fig. 3D). Both cleavage sites were within the predicted PRRRR sequence and the former confirmed the cleavage site found within the synthetic peptide (Fig. 3B). Thrombin did not cleave recombinant DBLγ6 domain from IT4var19 that was included as a negative control (Fig. 3E).

To determine the potential global relevance of the PPRRRR cleavage sites, we manually analyzed all published *var* genes from seven published parasite genotypes for the presence of PPRRRRLY. For each *var* gene, we ascertained the *var* group, presence of DBLδ1, and predicted binding phenotype (33) (see Table S1 in the supplemental material). Our search revealed that 135 of 368 *var* genes from *P. falciparum* contained the PPRRRRLY sequence*.* Of the 135 *P. falciparum* var genes, 130 contained a DBLδ1 with nearly all PPRRRRLY sequences found within this domain. Of those *var* gene products containing the sequence in question, 11% had an UpsA promoter, 59% were UpsB, and 30% were UpsC, compared to 19% UpsA, 60% UpsB, and 21% UpC *var* genes across the seven parasite genotypes, excluding the *var1CSA* pseudogene and the atypical type 3 *var* gene. The predicted binding phenotypes based on the CIDR1 domain type revealed 85% were CD36 binders (CIDRα2 to -6), 11% were EPCR binders (CIDRα1), and 4% (non-CIDRα) had an unknown binding phenotype, similar to their representation in the 3D7 genome reference isolate (84%, 11%, and 5%) (34). This suggests that although prevalent in 37% of all *var* genes, there is no selection bias for the PPRRRLY cleavage site between different *var* groups or binding phenotypes (Table 1; see Table S1).

**FIG 4:**
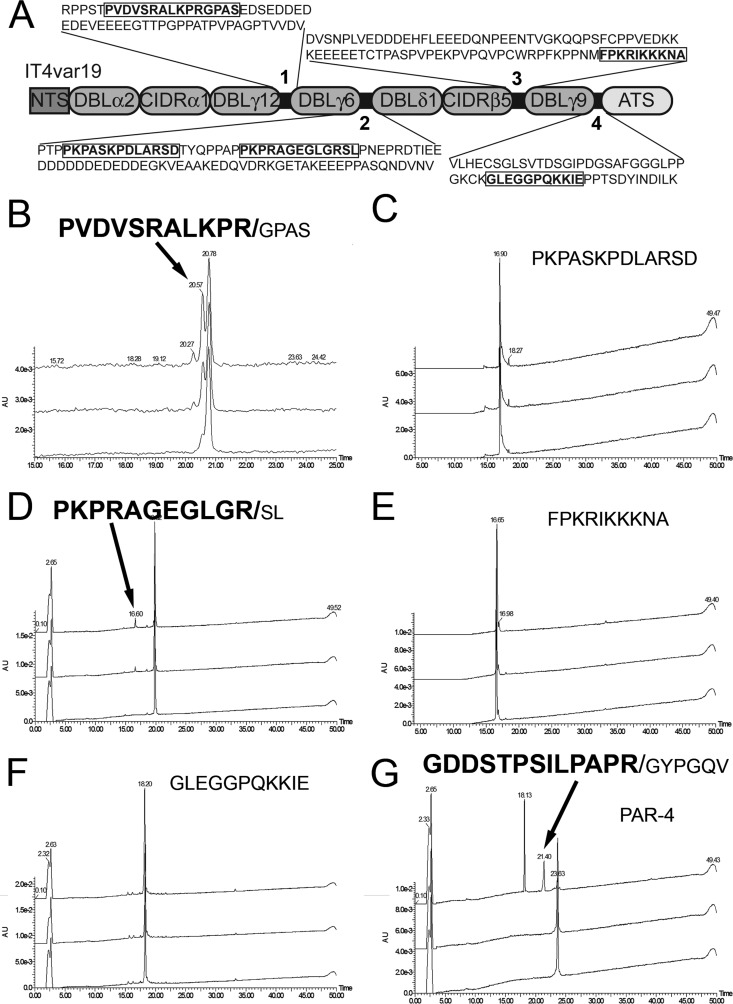
IT4var19 interdomains 1 and 2 are cleaved by thrombin. (A) Schematic of the IT4var19 interdomains with the full sequence of each domain (numbered 1 through 4). The sequences used to generate synthetic peptides for thrombin cleavage determination are indicated in boldface. (B to F) HPLC analysis of thrombin cleavage (25 nM) of the 5 peptides from IT4var19 interdomains. The fragments and cleavage sites (in boldface) corresponding to the HPLC peaks are indicated by arrows (*n* = 2). (G) Thrombin cleavage of protease-activated protein 4 (PAR4) was included as a positive control (*n* = 2). The molecular mass (Da) of each resulting fragment, as determined by TOF/TOF MS, is indicated above each peak.

We next focused on the most likely cut sites in the remaining extracellular IT4var19 protein selected manually from the four relatively short interdomain sequences (54 to 91 aa) as these sequences are predicted to have little secondary structure and would be accessible to thrombin (highlighted in Fig. 4A). Five highly purified (>95%) synthetic peptides from all four of the interdomains were incubated with thrombin for 30 min to 2 h, and the amounts and sites of cleavage were determined by HPLC and MALDI analysis (Fig. 4B to F). A positive-control peptide derived from the consensus thrombin cut site in PAR4 (35) cleaved at the expected site (Fig. 4E). Major cleavage of interdomain 1 following arginine 1236 (KPR/GPAS) and minor cleavage of interdomain 2 following arginine 1665 (GLGR/SL) were noted. No cleavage was found in the remaining peptides from domains 3 and 4.

### Blockade of thrombin exosite I reduces thrombin-induced endothelial permeability but preserves PfEMP1 cleavage.

**FIG 5:**
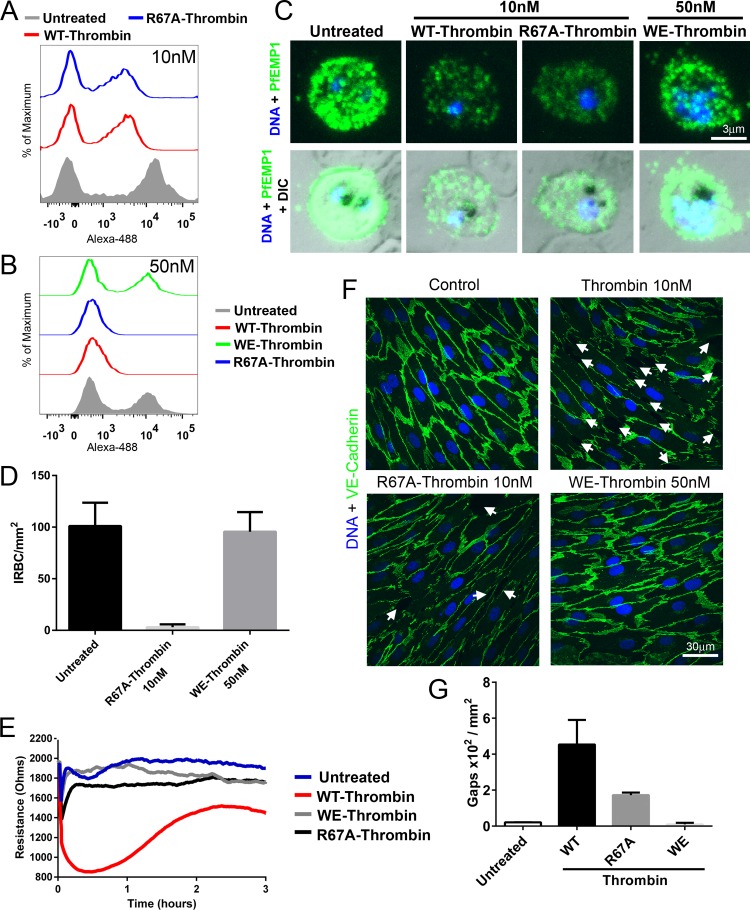
Effect of modified thrombins R67A-thrombin and WE-thrombin on PfEMP1 expression and endothelial permeability. Flow cytometry analysis of PfEMP1 expression on IRBC of IT4var19 treated with (A) 10 nM wild-type (WT) thrombin and R67A-thrombin or (B) 50 nM WT, R67A-, and WE-thrombin for 30 min at 37°C. Results are representative of 2 experiments. (C) Immunofluorescence microscopy of PfEMP1 expression on live IRBC from panel A. Images were taken at ×600 magnification. (D) Effect of 10 nM R67A- and 50 nM WE-thrombin on IRBC adhesion in the flow chamber assay. Results are presented as mean ± standard deviation (SD) (*n* = 2). **(**E) Changes in endothelial electrical resistance as monitored by the ECIS system in 3-day postconfluent HLMEC treated with 10 nM WT and R67A-thrombin and 50 nM WE-thrombin. Changes in resistance were monitored for 18 h at 37°C. A drop in electrical resistance was indicative of increased endothelial permeability. The results shown (from 0 to 3 h) are representative of 3 experiments. (F) Confocal immunofluorescence microscopy of HLMEC monolayers treated with 10 nM WT or R67A-thrombin or 50 nM WE-thrombin. Disruption of VE-cadherin expression and intercellular gap formation (indicated by white arrows) were induced by WT thrombin and to a much lesser degree by R67A-thrombin but not by WE-thrombin. Images are representative of 10 random fields examined at 600× in each of 2 independent experiments. (G) Quantification of microscopic changes seen in panel F. For each condition, three randomly selected microscopic fields at ×600 magnification were examined for the number of intercellular gaps of >5 µm in diameter and expressed as gaps × 10^2^ per mm^2^. The results shown are representative of 2 independent experiments.

The binding of substrates to thrombin and subsequent proteolysis are based on specific interactions with the active site, exosites I and II, and the presence of cofactors such as thrombomodulin which block the procoagulant exosite I of thrombin and mediate a switch in substrate preference (36). To further define the interaction between PfEMP1 and thrombin, the effect on cleavage of PfEMP1 from IT4var19 by two modified thrombins was studied. R67A-thrombin has a mutation at exosite 1, while W215A E217A-thrombin (WE-thrombin) has mutations in the terminal segment of the thrombin active site (37). PfEMP1 cleavage in response to the modified thrombins was monitored by flow cytometry, while endothelial permeability induced by the modified thrombins was determined as a reduction in transendothelial resistance (TER) measured by electric cell-substrate impedance sensing (ECIS). We found that R67A-thrombin at 10 and 50 nM had the same dose-dependent proteolytic effect on PfEMP1 of IT4var19 as thrombin (Fig. 5A to C). As a result, R67A-thrombin completely inhibited IRBC adhesion under flow conditions (Fig. 5D), while the effects on endothelial resistance (Fig. 5E) and disruption of the junctional protein VE-cadherin were reduced by 65% compared to thrombin (Fig. 5F and G). In contrast, WE-thrombin of up to 50 nM did not cleave IT4var19 PfEMP1 (Fig. 5B), did not inhibit IRBC adhesion (Fig. 5D), and had no effect on permeability (Fig. 5E) or junctional protein expression (Fig. 5F and G).

**FIG 6:**
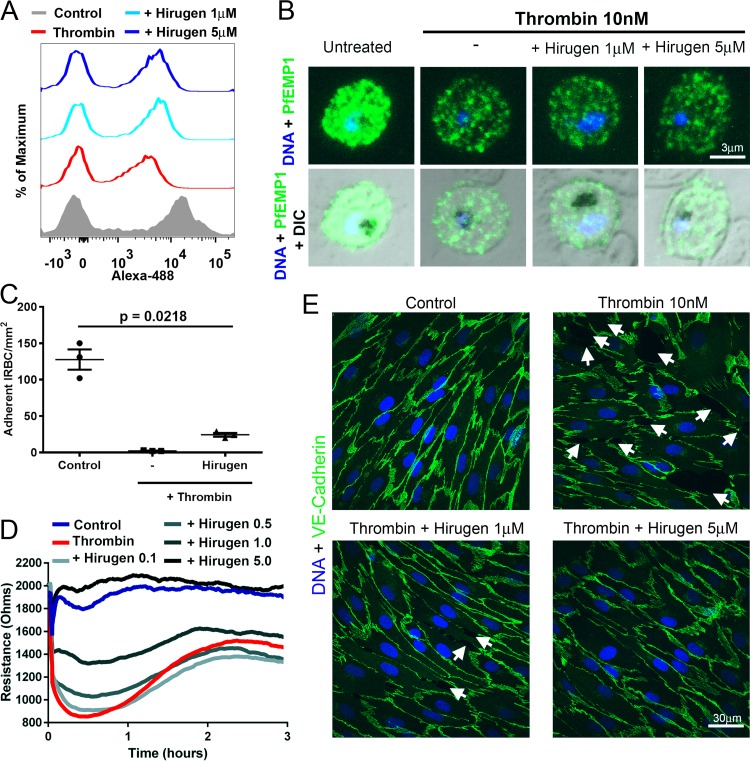
Hirugen inhibits endothelial barrier dysfunction but not IT4var19 cleavage induced by thrombin. (A) Flow cytometry analysis of PfEMP1 expression on IRBC of IT4var19 at the trophozoite stage treated with 10 nM thrombin with or without 1 and 5 µM hirugen for 30 min at 37°C. Results are representative of 2 experiments. (B) Immunofluorescence microscopy of IRBC from panel A. Images are representative of 10 randomly selected fields examined at ×600 magnification in each of 3 independent experiments. (C) Hirugen did not inhibit the effect of thrombin on IRBC adhesion under flow conditions. IRBC of IT4var19 were treated with thrombin with or without 1 µM hirugen for 30 min at 37°C before being used in a flow chamber assay. Results are expressed as mean ± SEM and were analyzed by ANOVA followed by *post hoc* multiple comparisons using Tukey’s test (*n* = 3). (D) Changes in endothelial resistance as monitored by ECIS of 3-day postconfluence HLMEC treated with 10 nM thrombin with or without 0.5 to 5 µM hirugen. The results shown are representative of 3 experiments. (E) Confocal immunofluorescence microscopy of HLMEC monolayers showing expression of VE-cadherin (green) and DNA (blue) in response to thrombin ± hirugen as in panel D. Images are representative of 10 random fields examined at 600× in each of 2 independent experiments.

We also tested the effect of a small sulfated peptide, hirugen (hirudin 54-65), that blocks exosite I on thrombin (38). In the presence of 1 and 5 µM hirugen, concentrations that do not inhibit endothelial APC generation (39), PfEMP1 cleavage was reduced by 45% and 40%, respectively, compared to thrombin alone (Fig. 6A and B). However, the reduction in ligand expression compared to untreated IRBC was sufficient to reduce adhesion of IRBC under flow conditions by 81% ± 0.3% (*n* = 3) (Fig. 6C). Hirugen at 1 µM also inhibited thrombin-induced permeability by 49% ± 7% (*n* = 3; *P* = 0.013), while at 5 µM, the inhibition of thrombin activity on permeability (Fig. 6D) and disruption of VE-cadherin expression (Fig. 6E) were complete.

### Wild-type thrombin and R67A-thrombin but not hirugen-treated or WE-thrombin detach adherent IRBC.

**FIG 7:**
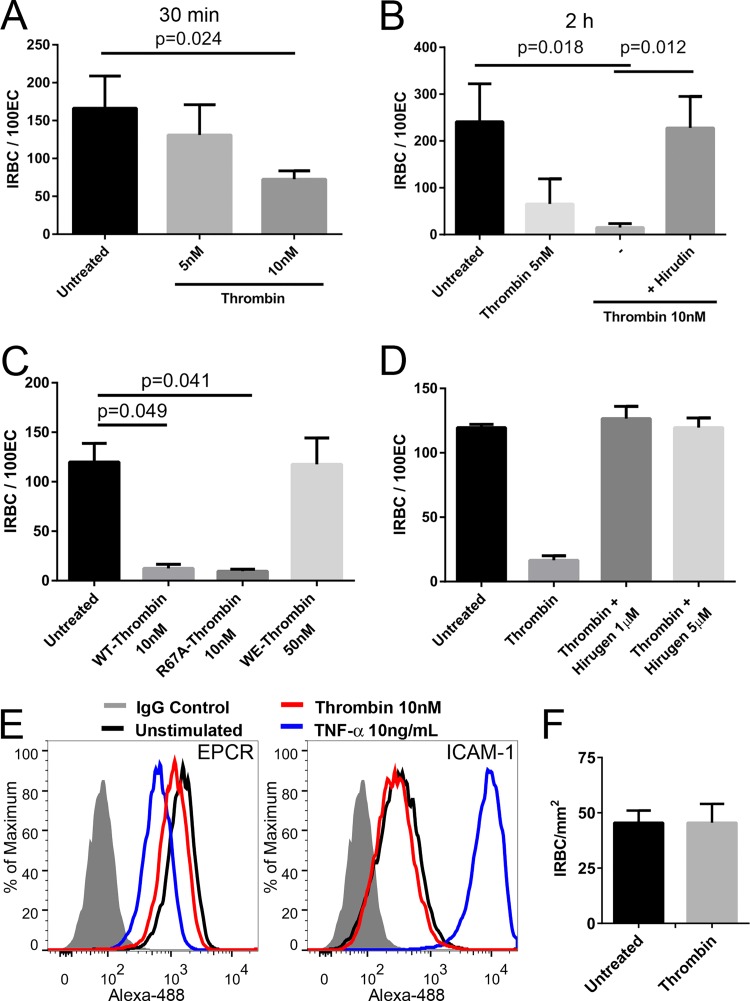
Thrombin detaches adherent IRBC from HLMEC. (A) Detachment of adherent IRBC from HLMEC monolayers following thrombin treatment (5 or 10 nM) of cocultures for 30 min at 37°C (*n* = 3). (B) Detachment of adherent IRBC from HLMEC monolayers following thrombin treatment (5 or 10 nM) of cocultures for 2 h at 37°C (*n* = 4). (C) R67A- but not WE-thrombin detaches adherent IRBC. Results are shown as mean ± SEM (*n* = 3) and were analyzed by ANOVA followed by *post hoc* multiple comparisons using Tukey’s test. (D) Hirugen inhibited the effect of thrombin on detachment of adherent IRBC. Results are presented as mean ± SD (*n* = 2). (E) Thrombin treatment had no effect on ICAM-1 or EPCR expression. (F) Lack of effect on adhesion under flow conditions on HLMEC pretreated with 10 nM thrombin for 30 min.

To determine if thrombin could detach already adherent IRBC, IRBC of IT4var19 were allowed to adhere for 1 h to a monolayer of HLMEC prior to the addition of 5 or 10 nM thrombin for 30 min and 2 h. After nonadherent IRBC were removed by gravity sedimentation, the number of adherent IRBC per 100 endothelial cells (EC) was quantified. The results showed a time- and concentration-dependent effect of thrombin. The number of adherent cells was significantly reduced by 55% ± 6% and 94% ± 3% following incubation with 10 nM thrombin for 30 min and 2 h, respectively (*n* = 4; *P* = 0.024 and 0.018) (Fig. 7A and B. The near total absence of adherent IRBC on the monolayers after 2 h of thrombin treatment is consistent with both the detachment of already adherent IRBC and inhibition of further adhesion. The effect of thrombin was completely abrogated by preincubation with 100 nM hirudin. Similar effects were shown between wild-type thrombin and R67A-thrombin at 10 nM but not WE-thrombin at 50 nM (Fig. 7C). In contrast, pretreatment of thrombin with hirugen inhibited its ability to detach IRBC adhesion (Fig. 7D). The discrepancy between the effect of hirugen on adhesion under flow (Fig. 6C) and the reversal of binding studies done under static conditions (Fig. 7) could be due to the greater sensitivity of the flow adhesion assay to reductions in PfEMP1 expression.

To investigate whether thrombin may also cleave parasite adhesion receptors from the surface of endothelial cells, HLMEC were pretreated with 10 nM thrombin prior to a flow chamber assay. Thrombin did not modify surface levels of two known parasite cytoadhesion receptors, intercellular adhesion molecule 1 (ICAM-1) and EPCR (Fig. 7E). Conversely, the proinflammatory cytokine tumor necrosis factor alpha (TNF-α) led to a partial reduction of EPCR expression and an approximately 50-fold increase in ICAM-1 expression, as expected (Fig. 7E). Thrombin pretreatment of HLMEC also had no effect on IRBC adhesion under flow conditions (Fig. 7F).

### Presence of microvascular thrombin inversely correlates with IRBC sequestration in pediatric cerebral malaria.

**FIG 8:**
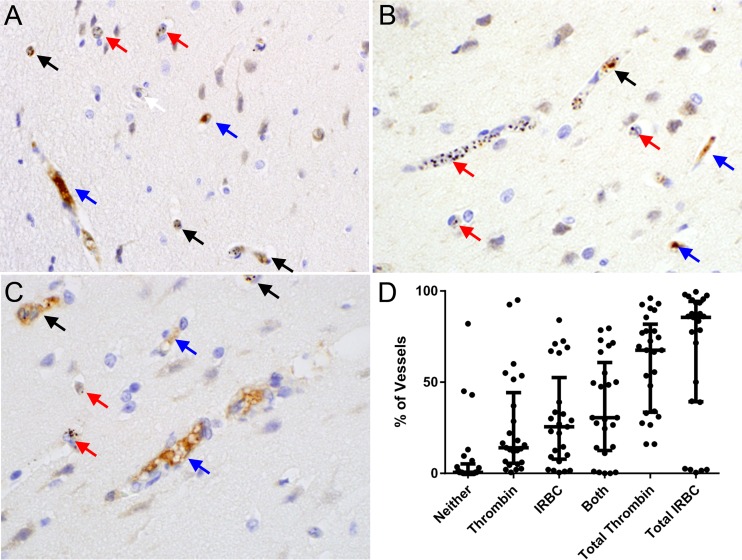
Thrombin staining correlates inversely with parasite sequestration in brain microvascular endothelium in pediatric patients with cerebral malaria. (A to C) Immunohistochemistry of brain sections from pediatric patients with cerebral malaria demonstrating thrombin staining (dark brown) and sequestered parasites in microvessels. Cerebral microvessels were scored as demonstrating sequestration alone (red arrows), thrombin alone (blue arrows), both (black arrows), or neither (white arrows). Quantitation showed an overall negative correlation between sequestration and thrombin staining (*r*_ф_ = −0.20; 95% CI, −0.175 to −0.227; *P* < 0.001). (D) Cumulative results for 200 cerebral microvessels in each of 25 patients with CM1 to 9 assessed blind independently by two microscopists.

Tissue factor production by endothelial cells has been shown to be induced at sites of IRBC adhesion (17), initiating the extrinsic coagulation pathway. To determine if thrombin is detectable in cerebral microvessels and whether the presence of thrombin bears any relationship to IRBC sequestration, sections of postmortem brain tissue were examined by immunohistochemistry (IHC). The study population was a previously defined convenience sample of pediatric patients representing low to high sequestration in an ongoing study aimed at defining the clinicopathological correlation of cerebral malaria (CM) (D. A. Milner, Jr., K. B. Seydel, and T. E. Taylor, unpublished data). The patient set was therefore not selected specifically for this study, nor was the inclusion or exclusion of cases dependent on any criteria related to the presence of thrombin and/or sequestration. CM was defined as the presence of IRBC or pigment clumps in >21% of cerebral microvessels (40). Two hundred randomly selected vessels from each of the 25 patients were scored for the presence or absence of thrombin and sequestered IRBC (Table 2). Representative images of Thr^+^/Seq^+^, Thr^+^/Seq^−^, Thr^−^/Seq^+^, and Thr^−^/Seq^−^ vessels are shown in Fig. 8A to C.

**TABLE 2:** Quantitation of sequestration and thrombin staining in cerebral microvessels from pediatric patients with cerebral malaria

Case no.	Diagnosis^a^	% PV^b^	No. of microvessels with thrombin/sequestration^c^				OR	eOR^d^	*P* value^e^
			Neither	Thrombin	IRBC	Both			
37	CM1	26.2	14	186	1	0		0.04	0.075
100	CM1	37.0	19	103	25	53	0.39	0.40	**0.005**
99	CM1	60.9	7	36	58	99	0.33	0.37	**0.007**
95	CM1	84.9	0	9	51	140		0.27	0.066
1	CM1	85.4	26	74	78	22	0.10	0.10	**<0.001**
96	CM2	35.6	3	26	142	29	0.02	0.03	**<0.001**
85	CM2	43.1	0	13	132	55		0.03	**<0.001**
36	CM2	45.0	0	33	67	100		0.04	**<0.001**
55	CM2	48.1	0	25	65	110		0.06	**<0.001**
78	CM2	48.1	0	57	46	97		0.04	**<0.001**
94	CM2	51.3	1	12	54	133	0.21	0.37	0.083
60	CM2	53.3	0	10	44	146		0.30	0.078
26	CM2	56.3	1	27	13	159	0.45	0.81	0.388
83	CM2	63.1	1	44	60	95	0.04	0.07	**<0.001**
32	CM2	77.2	0	28	29	143		0.17	**0.009**
69	CM2	80.7	0	1	138	61		0.22	0.310
35	CM2	83.7	0	4	168	28		0.03	**0.001**
6	CM2	85.7	2	4	145	49	0.17	0.21	**0.044**
66	CM2	87.7	1	120	20	59	0.02	0.05	**<0.001**
98	CM2	91.1	1	24	18	157	0.36	0.67	0.279
28	CM2	96.2	0	5	134	61		0.08	**0.004**
58	CM3	4.3	6	190	2	2	0.03	0.04	**0.008**
31	CM3	4.5	164	31	4	1	1.32	2.06	0.586
7	CM6	4.8	90	107	3	0		0.21	0.099
10	CM9	12.7	86	110	3	1	0.26	0.39	0.233
Total			422	1,279	1,500	1,800	0.40	0.41	<0.0001

a Diagnosis: CM1, sequestration only; CM2, sequestration and fibrin deposition; CM3, coma with no malarial pathology in brain; CM6, severe malarial anemia; CM9, pneumonia and incidental parasitemia.

b % PV is the percentage of parasitized cerebral microvessels in autopsy tissue sections used for diagnosis of CM (40).

c Two hundred microvessels from each patient were scored for the presence of thrombin, sequestration (IRBC), neither, or both.

d Cells with 0 counts made the odds ratio (OR) for those samples impossible to calculate. Estimated OR (eOR) were computed by adding 1 to all cells with a 0 count.

e Boldface P values are <0.05.

Quantitation of the vessels from all 25 cases demonstrated thrombin staining alone in 14% (median) of cerebral microvessels (interquartile range [IQR], 5 to 44%), IRBC alone in 25% (median; IQR, 8 to 52%), and the presence of IRBC and thrombin together in 30% (median; IQR, 12 to 61%) (Fig. 8D; Table 2). When the results were expressed as an odds ratio (OR), where any number that is <1.0 demonstrates an inverse correlation between sequestration and thrombin, the OR for all individual cases was less than 1, with the exception of 1 case (range, 0.02 to 0.45; *P* < 0.001 to 0.388). A similar finding was seen in the estimated OR. The summary statistic for all vessels showed an actual OR and estimated OR (eOR) of 0.40 and 0.41, with a *P* value of <0.0001 for both. Overall, a negative correlation between sequestration and thrombin staining was confirmed by the phi coefficient (*r*_φ_) for the 21 cerebral malaria cases (*r*_φ_ = −0.31; 95% confidence interval [CI], −0.29 to −0.36; *P* < 0.001) or all 25 cases (*r*_ф_ = −0.20; 95% CI, −0.175 to −0.227; *P* < 0.001).

## DISCUSSION

Thrombin, the pivotal molecule in fibrin clot and APC generation, is a highly selective serine protease with well-characterized human substrates involved in coagulation (factors V, VIII, XI, and XIII; protein C; and fibrinogen) or endothelial barrier function (PAR1, -3, and -4). The recent characterization of the thrombin consensus cleavage site of Pro–Arg-(Ala-Gly-Ser-Thr)-(not Asp-Glu)-Arg (PRXXR) has led to the prediction of numerous additional human thrombin substrates involved in cell adhesion (e.g., the integrin α_V_), central nervous system development, and circulatory homeostasis (32). Among pathogens, only pallilysin from *Treponema pallidum*, the causative agent of syphilis, is known to be cleaved by thrombin from a proenzyme into an active metalloprotease allowing the spirochete to degrade fibrin clots and propagate infection (41). To date, no studies have investigated the impact of thrombin on *P. falciparum* sequestration, the key pathogenic process in severe malaria.

In this study, we demonstrate that at physiologically relevant concentrations, thrombin cleaves the IRBC cytoadherent ligand PfEMP1 from the surface of IRBC in a dose-dependent fashion and impairs their adhesion to microvascular endothelium under flow conditions or even detaches already adherent IRBC from endothelial monolayers *in vitro*. Cleavage was complete at 50 nM thrombin, but the 75% reduction of PfEMP1 expression seen at 10 nM was sufficient to result in a functional change, an observation that implies that there may be a threshold required for the density of receptor and ligand molecules and/or avidity of receptor-ligand interaction to mediate adhesion, particularly under physiological flow conditions. Whether the reduction of expression/function is due to partial cleavage of PfEMP1 on some IRBC or a decrease in the absolute number of PfEMP1 molecules that were completely removed is not addressed by our data. The proteolytic effect is seen in laboratory-selected lines as well as clinical isolates. These results strongly suggest that thrombin cleavage is independent of the adhesive phenotype of the parasites, since both CD36 and EPCR binding parasite lines were sensitive to cleavage.

Using synthetic peptides and recombinant protein, we mapped putative thrombin cleavage sites to the DBLδ1 domain and interdomains 1 and 2 in the IT4var19 PfEMP1. Cleavage of DBLδ1 that is proximal to most of the PfEMP1 domains would explain the near complete inhibition of adhesion on both HLMEC and HDMEC. The interdomain 1 and 2 cleavage sites may also contribute to the inhibition of adhesion, as they are both proximal to the semiconserved head structure that has been shown to mediate adhesion to multiple adhesion molecules (34). The true function of these interdomains is unclear, although they do contain conserved elements and are predicted to have low secondary structure (33), possibly rendering them accessible to serum proteases. However, it should be noted that the DBLδ1 and interdomain predicted cut sites on IT4var19 that we studied are by no means exhaustive, as we found an additional 34 high-probability cut sites using established algorithms in 13 of the remaining 14 *var* genes examined in Table 1 that represented parasites of diverse adhesive phenotypes. Therefore, more PfEMP1 variants than IT4var19 will need to be studied using native or full-length PfEMP1 to comprehensively identify the thrombin cleavage sites and to determine the kinetics and efficiency of the cleavage. The ultimate functional importance of potential thrombin cut sites is likely to depend on the location, abundance, affinity, and accessibility of the cleavage sequences to thrombin.

**FIG 9:**
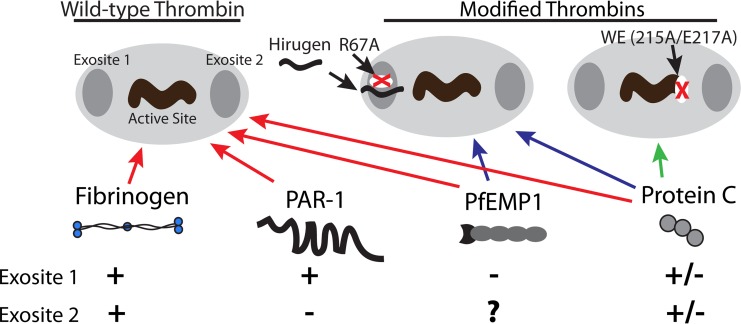
Schematic representation of the binding of PfEMP1 and natural ligands to wild-type and modified thrombins. Proteolytic activity of thrombin is governed by three key binding sites, including exosites I and II and the enzymatic active site. Exosite I is mutated in R67A-thrombin or blocked by hirugen, and the active site itself is mutated in WE-thrombin. Breakdown of fibrinogen requires interaction with all 3 components of wild-type thrombin. Proteolysis of PAR1 requires interaction with exosite I but not II. Protein C itself does not bind exosite I or II, but activation is enhanced by binding of the cofactor thrombomodulin to exosites I and II. PfEMP1 cleavage does not require interaction with exosite I, while the requirement for exosite II is not known.

To further delineate the molecular interaction between PfEMP1 and thrombin, we tested the proteolytic activity of two modified thrombins toward PfEMP1. A schematic model is shown in Fig. 9. We found that the active site of thrombin is of critical importance as WE-thrombin has no proteolytic activity toward PfEMP1. This result is not unexpected in view of the compromised activity of this mutant except toward protein C in the presence of thrombomodulin (42). In contrast, R67A-thrombin has proteolytic activity toward PfEMP1 comparable to that of thrombin, suggesting that exosite I is not required for PfEMP1 cleavage. The lack of involvement of exosite I in PfEMP1 cleavage is supported by our results using hirugen, a competitive exosite I inhibitor. These results are noteworthy given that thrombin activity toward many physiological substrates in the proinflammatory and coagulation cascades specifically requires exosite I and, to some extent, exosite II interaction. Any beneficial effect of thrombin that only depends on the active site opens significant opportunities in therapeutic intervention for severe malaria. Several thrombin mutants like R67A-thrombin have been engineered with compromised exosite I activity but normal activity toward chromogenic substrates that only bind to the active site (37). R67A-thrombin would be an attractive therapeutic candidate because it has 100-fold-reduced activity toward fibrinogen and PAR1 but only a10-fold-reduced activity toward protein C (37). Although R67A-thrombin binds to PAR1 with 10^5^-fold-less affinity than native thrombin (43), and therefore would be ineffective as a competitive inhibitor to block endogenous thrombin activation of PAR1, it has been engineered to have limited proinflammatory activity while retaining selectivity for protein C activation, and as shown here, it retains potential PfEMP1 cleavage function. On the other hand, hirugen, a small molecule that inhibits substrate binding to exosite I as does R67A-thrombin, could shift the activity of endogenous thrombin toward beneficial effects. Hirugen was effective in ameliorating the disruptive effect of thrombin on endothelial barrier integrity while preserving sufficient proteolytic cleavage of PfEMP1 to inhibit adhesion under shear stress. It is interesting to note that the effect of hirugen on exosite 1 is similar to that of soluble thrombomodulin, which is currently undergoing clinical trials for the treatment of sepsis and disseminated intravascular coagulation (44). While antimalarial drugs kill parasites, they may not immediately reverse pathophysiological processes in occluded vessels. Therefore, we can envision combining modified thrombin-based approaches with antimalarial drugs to achieve both rapid parasite killing and release of sequestered IRBC.

Whether thrombin cleavage of PfEMP1 occurs *in vivo* remains to be determined. In the absence of an adequate animal model for *P. falciparum* sequestration, which would have allowed for the examination of PfEMP1 cleavage in the complex milieu of an acute infection, the presence of thrombin in cerebral microvessels and the inverse correlation with sequestration in pediatric CM autopsies suggest that thrombin could cleave PfEMP1 *in vivo*. The extent to which this process occurs would depend on the local thrombin concentration at the site of cytoadhesion and the shear stress in the microvessels, as well as the susceptibility of the particular PfEMP1 variants expressed by the parasite isolates. At sites of lower thrombin production, IRBC may be detached or prevented from adhering in the absence or presence of coagulation abnormalities. We in fact demonstrated that surface-expressed PfEMP1 on IRBC is cleaved by thrombin at a concentration that is far lower than the levels observed during fibrinogenesis *in vivo* (200 to 300 nM). This process may occur in patients who do not go on to develop severe disease and hence would not be examined at autopsy. Indeed, there is evidence that thrombin activation markers, including d-dimer and fibrin degradation products, are significantly elevated in uncomplicated malaria compared to healthy controls (45). At sites of higher thrombin production, thrombin cleavage sites may have been retained in the highly variant PfEMP1 family as a “steam valve-like” mechanism to release sequestered IRBC. This would favor host survival and parasite transmission. However, thrombin activation also leads to the generation of excessive coagulation factors that are important mediators of inflammation, as well as promoting microvascular thrombosis. In addition, the activation of PAR1 by thrombin results in the generation of proinflammatory cytokines and disruption of barrier integrity. These detrimental effects of thrombin will contribute to the pathology of severe malaria in addition to its effect on sequestration. This scenario is similar to that with a number of inflammatory mediators, such as tumor necrosis factor, which at low levels may be beneficial to the host in stimulating innate immune responses. However, an uncontrolled or exaggerated cytokine response would lead to immunopathology and demise of the host. This makes our proposed therapy with modified thrombins (R67A) and thrombin modulators (hirugen) attractive as they can serve to shift thrombin cleavage events toward protein C activation and away from PAR1 and fibrinogen activation, while both preserve the ability to cleave PfEMP1.

Our proposal is not entirely in contrast to that of Moxon et al. (16), who reported a correlation between high levels of thrombin and disease severity and the colocalization of thrombin clots with sequestered IRBC in cerebral microvessels. In the Moxon study, circulating levels of thrombin were deduced from the thrombin/antithrombin III (TAT) complex. As the complex is inactive, this measure may more accurately reflect cumulative thrombin production rather than active thrombin at a given time point. The level of circulating thrombin may also be different from that at a local site. Among the 25 patients in our convenience samples, a significant number of cerebral microvessels (median, 30%) had both IRBC and thrombin and, presumably, fibrin clots. An additional confounding factor is the possibility that IRBC with cleaved PfEMP1 may remain trapped within microvascular fibrin deposits. It should also be pointed out that fibrin deposition is not a universal feature of cerebral malaria. There is a subgroup of patients designated CM1 in whom sequestration occurs in the absence of fibrin clots. Taken together, these findings suggest a complex interplay between cytoadherence, thrombin activation, and clot formation *in vivo* that may not be completely explained by autopsy findings.

To understand whether thrombin may modify parasite adhesion receptors on endothelial cells, the proteolytic effect of thrombin on endothelial receptors was examined. In preliminary experiments, adhesion of IT4var19 to HLMEC monolayers preincubated with 10 nM thrombin for 30 min was not reduced, and thrombin had no effect on endothelial ICAM-1 or EPCR expression. However, whether higher doses of thrombin or longer duration would have a different effect on HLMEC and other microvascular endothelium remains to be determined. Wild-type thrombin has been reported to induce adhesion molecules such as P-selectin and ICAM-1 on human umbilical vein endothelial cells within 15 to 30 min (46). As well, other molecules involved in cell adhesion and parasite binding are now predicted to be thrombin substrates (e.g., integrins [47]), so that the modulation of their expression by thrombin will also need to be determined. Based on the finding that all 7 recombinant IT4var19 DBL or CIDR domains bind to different types of microvascular endothelial cells via many as yet unidentified receptor molecules (26), targeting specific endothelial receptors would appear to be a very complex and perhaps unattainable goal compared to targeting a relatively common proteolytic cleavage site in the variant parasite adhesin family. More importantly, what we observed for thrombin may be a broader phenomenon involving other serum proteases that are increased in patients with severe malaria (16, 48). Our study suggests that modified thrombins may constitute a new approach to antiadhesive therapy.

## MATERIALS AND METHODS

### Ethics statement.

Cryopreserved clinical parasite isolates obtained from adult Thai patients with acute falciparum malaria were obtained at the Hospital for Tropical Diseases, Bangkok, Thailand (27). The collection of specimens was approved by the Ethics Committee of the Faculty of Tropical Medicine, Mahidol University. Informed consent was obtained from all patients and/or relatives according to the Declaration of Helsinki. Autopsy specimens from patients with cerebral malaria and noncerebral controls were obtained in an ongoing study (D.A.M.) focused on understanding the dynamics of sequestration. The study was approved by the ethics committees at the University of Liverpool, Michigan State University, and the University of Malaŵi College of Medicine. Informed consent was obtained from all patients and/or relatives according to the Declaration of Helsinki. Discarded human foreskins were collected for the isolation of endothelial cells for this study with written informed consent of the parents. Normal red blood cells were collected from adult volunteer donors with their written informed consent. Both protocols were reviewed and approved by the Conjoint Ethics Board of Alberta Health Services and The University of Calgary, Alberta, Canada.

### Tissue culture and other reagents.

Unless otherwise specified, all tissue culture reagents were obtained from Invitrogen Life Technologies Canada, Inc. (Burlington, ON, Canada), and chemical reagents were purchased from Sigma-Aldrich, Co. (St. Louis, MO). The thrombin inhibitor recombinant hirudin was purchased from Hyphen Biomed (Neuville-sur-Oise, France). The modified thrombins WE-thrombin and R67A-thrombin were produced and purified as described previously (36). Endothelial basal medium (EBM) and supplements were purchased from Lonza Group, Ltd. (Walkersville, MD).

### Parasites.

The parasite lines IT4var19, IT4var07, IT4var01, and IT4var11 were isolated from the parental strain IT4/25/5, as well as HB3var03 from HB3, by limiting dilution cloning (26). The expression of PfEMP1 in the five parasite lines was monitored by flow cytometry with *var* gene-specific polyclonal antibodies and/or *var* gene transcription profiling (25).

### Microvascular endothelial cells.

Primary human lung microvascular endothelial cells (HLMEC) were purchased from Lonza (21). Primary human dermal microvascular endothelial cells (HDMEC) were harvested and purified from discarded neonatal human foreskins (45). An immortalized brain endothelial cell line (THBMEC) was used (29).

### Flow chamber assay.

IRBC-endothelial cell interactions at fluid shear stresses approximating those in the microvasculature were studied using a parallel-plate flow chamber as described previously (27). Frozen aliquots of parasites were thawed and cultured for 24 to 30 h at 37°C and 95% N_2_–5% CO_2_ with RPMI plus 10% pooled human AB serum until the late trophozoite/early schizont stage as determined by light microscopy. Thawed parasites were used in experiments for 2 to 4 cycles, after which they were discarded. At the time of experimentation, a 1% IRBC suspension at 4 to 5% parasitemia pretreated with thrombin in serum-free medium for 30 min was infused over confluent HLMEC or HDMEC monolayers at 1 dyne/cm^2^, which allowed for optimal visualization of the adhesive interactions in real time. Experiments were recorded and analyzed off-line. An adherent IRBC was defined as one that remained attached for >10 s. Results are expressed as the mean number of adherent IRBC/mm^2^ in 4 randomly selected fields of view (200×) at the end of a 7-min infusion.

### Static binding and detachment assay.

Static binding assays for the parasite lines IT4var19, IT4var07, and HB3var03 on THBMEC were performed as described previously (29). For the detachment assay, IRBC at 1% hematocrit and 5 to 6% parasitemia in serum-free EBM were allowed to adhere to monolayers of HLMEC on glass coverslips for 1 h at 37°C before the addition of 5 or 10 nM thrombin for 30 min or 2 h. Thrombin was added gently to the side of the chambers and allowed to slowly diffuse through the medium to avoid dislodging already adherent IRBC or activating endothelial cells. The coverslips were then washed by gravity. Air-dried and methanol-fixed coverslips were stained with Field stain, and the number of adherent IRBC under each experimental condition was quantified at a ×1,000 magnification by counting along the diameter of the coverslip (≈300 to 400 endothelial cells). The data are expressed as the number of adherent IRBC/100 EC.

### Recombinant PfEMP1 protein/peptide synthesis and cleavage analysis.

Recombinant DBLδ1 and DBLγ6 domains from IT4var19 were generated as previously described (26). Peptides from IT4var19 interdomains, DBLδ1, and PAR4 were made by solid-phase synthesis on a Rink amide resin and purified to >95% by high-performance liquid chromatography (HPLC). One hundred micromoles of purified peptide or 5 µg recombinant protein was exposed to thrombin at 25 or 250 nM, respectively, for 30 min to 2 h at 37°C. Cleavage products were analyzed by HPLC and matrix-assisted laser desorption-ionization (MALDI) for peptide cleavage products or MALDI alone for recombinant protein cleavage products.

### Flow cytometry analysis of IRBC.

Trophozoite-stage IRBC of IT4var19, IT4var07, or HB3var03 were incubated for 45 min at 4°C with rabbit polyclonal antibody against the corresponding DBLα domain specific to each *var* gene at a 1:20 dilution. Antibody labeling was detected with goat anti-rabbit IgG-Alexa 488 (Molecular Probes) at a 1:400 dilution for 30 min. Infected erythrocyte nuclei were detected with ethidium bromide (Invitrogen) at a 1:500 dilution added with the secondary antibody. Stained cells were washed in phosphate-buffered saline (PBS) and analyzed on an LSRII fluorescence-activated cell sorter (FACS) machine (BD Biosciences). Analysis was performed using FlowJo 8 (Tree Star, Inc., Ashland, OR).

### Immunofluorescence microscopy of IRBC.

Live IRBC stained as described above for flow cytometry analysis were used to prepare blood smears on glass slides. Air-dried smears were fixed in ice-cold 90% acetone–10% methanol at −20°C for 10 min. Coverslips were mounted with ProLong Gold and imaged by wide-field immunofluorescence. Images were acquired using Openlab 5.0.2 (Improvision, Lexington, MA) on an Olympus IX70-S8F2 inverted microscope (Center Valley, PA) using a cooled charge-coupled device (CCD) Retiga EXi camera from Q Imaging (Vancouver, BC, Canada). All images were taken with a 60× objective under oil immersion, where each field is equal to 224.2 by 170.8 µm (1,360 by 1,036 pixels).

### Confocal immunofluorescence microscopy.

Endothelial cells were seeded on gelatin-coated glass coverslips for characterization of junctions as previously described. All experiments were done at 48 to 72 h postconfluence with endothelial cells seeded at 5 × 10^4^ cells/cm^2^. A monoclonal anti-VE-cadherin antibody (2 µg/ml; clone F-8 [Santa Cruz Biotech, Dallas, TX]) was used for characterization of endothelial cell junctions. Alexa 488-labeled secondary antibody was added at 1:500 for an hour. After washing, the coverslips were imaged using an Olympus IX81 FV100 laser scanning confocal microscope with 60× OPlanFLN oil objective NA 1.42 (Olympus Canada, Inc., Toronto, ON, Canada). Images were acquired at 800 by 800 pixels (212 µm by 212 µm) and 20 to 30 0.5-µm stacks at 2× Nyquist sampling using FV1000 acquisition software.

Image analysis was done using ImageJ v1.48 (NIH, Rockford, MD). Gaps in HLMEC were quantified by randomly selecting 3 microscopic fields for each condition in each experiment. A gap was defined as a single area larger than 5 µm in diameter devoid of cells (21). Results obtained from 2 independent experiments were expressed as gaps × 10^2^ per mm^2^.

### Endothelial resistance.

Real-time change in endothelial monolayer resistance as a measure of endothelial barrier function was determined using the ECIS system (Applied Biophysics, Troy, NY) according to the instructions of the manufacturer. In brief, 5 × 10^4^ HLMEC were plated on a small gold electrode (well size, 0.8 cm^2^) precoated for 10 min with 10 µM l-cysteine. When the cells were 72 h postconfluent, the medium was changed to serum-free EBM and allowed to equilibrate for 2 h. Thrombin, modified thrombin, or thrombin with or without the inhibitors hirudin and hirugen was then added in 25-µl volumes. Changes in electrical resistance were monitored continuously for up to 18 h after the thrombin challenge.

### Brain histology and thrombin staining.

Immunohistochemical staining for thrombin was performed on 4-µm sections of formalin-fixed paraffin-embedded tissues by standard methods after antigen retrieval using a thrombin-specific monoclonal antibody (MAb) (clone 5G9 [Abcam]) at a 1:100 dilution. Slides were counterstained with hematoxylin and were independently scored blind by two microscopists for the presence of thrombin (Thr) staining and/or sequestration (Seq), creating four categories of vessels: Thr^+^/Seq^+^, Thr^+^/Seq^−^, Thr^−^/Seq^+^, and Thr^−^/Seq^−^.

### Statistical analysis.

Statistical analysis of *in vitro* studies was performed using GraphPad Prism (version 6; GraphPad Software, Inc., La Jolla, CA). Data are expressed as the mean ± standard error of the mean (SEM) unless otherwise stated. All data were compared using analysis of variance (ANOVA) followed by *post hoc* multiple comparisons using Tukey’s test unless otherwise specified. Phi correlation (*r*_ф_) and odds ratio (OR) analysis were used for clinical data. *P* values of ≤0.05 were considered statistically significant.

## SUPPLEMENTAL MATERIAL

Table S1  Analysis of *P. falciparum var* genes for the presence of the putative thrombin cleavage site. All published *P. falciparum var* genes from seven published parasite genotypes (33) were analyzed for the presence of the putative thrombin cleavage site PPRRRRLY. For each *var* gene, the *var* group (UPS), presence of the DBLδ1 domain, and predicted binding phenotype were ascertained. Table S1, PDF file, 0.4 MB
