# Supplementary material for: Thrombin Cleavage of Plasmodium falciparum Erythrocyte Membrane Protein 1 Inhibits Cytoadherence
Source: mBio. 2016 Sep 13;7(5):e01120-16. doi: 10.1128/mBio.01120-16 (PMC5021802; doi:10.1128/mBio.01120-16)
Supplement: Table S1 — Analysis of P. falciparum var genes for the presence of the putative thrombin cleavage site. All published P. falciparum var genes from seven published parasite genotypes (33) were analyzed for the presence of the putative thrombin cleavage site PPRRRRLY. For each var gene, the var group (UPS), presence of the DBLδ1 domain, and predicted binding phenotype were ascertained. [file mbo004162986st1.pdf]

Table S1. Analysis of *P. falciparum var* genes for the presence of the putative thrombin cleavage site. All published *P. falciparum var* genes from seven published parasite genotypes (33) were analyzed for the presence of the putative thrombin cleavage site PPRRRRLY. For each *var* gene, the *var* group (UPS), presence of the DBLδ1 domain, and predicted binding phenotype were ascertained.

PPRRRRLY	DBLδ1	UPS	Binding Phenotype	Domain Content
		**DD2**		
var01a	1	B	CD36	DBLα0.6 CIDRα3.1 DBLβ5 DBLγ5 DBLδ1 CIDRβ1
var01b	1	B	CD36	DBLα0.6 CIDRα3.1 DBLβ5 DBLγ5 DBLδ1 CIDRβ1
var07	1	C	CD36	DBLα0.16 CIDRα2.3 DBLδ1 CIDRβ5
var09b	1	A	OTHER	DBLα1.5 CIDRδ2 DBLγ11 DBLβ7 DBLδ1 CIDRβ1
var10	1	C	CD36	DBLα0.15 CIDRα3.2 DBLδ1 CIDRβ1
var11	1	B	CD36	DBLα0.15 CIDRα3.2 DBLδ1 CIDRβ1
var12	1	C	CD36	DBLα0.24 CIDRα3.3 DBLδ1 CIDRγ6
var13	1	B	CD36	DBLα0.8 CIDRα3.5 DBLβ8 DBLδ1 CIDRβ1
var19	1	B	CD36	DBLα0.10 CIDRα2.2 DBLδ1 CIDRγ9 DBLζ6 DBLε6
var20	1	B	CD36	DBLα0.9 CIDRα2.7 DBLδ1 CIDRβ6
var24	1	B	CD36	DBLα0.15 CIDRα3.2 DBLβ5 DBLδ1 CIDRγ1 DBLγ3 DBLζ4
var26	1	C	CD36	DBLα0.19 CIDRα2.3 DBLδ1 CIDRβ5
var29	1	B	CD36	DBLα0.16 CIDRα3.4 DBLδ1 CIDRβ1 DBLγ10
var31	1	B	CD36	DBLα0.5 CIDRα2.6 DBLδ1 CIDRβ1
var32	1	A	EPCR	DBLα1.7 CIDRα1.4 DBLβ1 DBLβ6 DBLγ11 DBLδ1 CIDRγ5
var33	1	B	CD36	DBLα0.9 CIDRα2.1 DBLδ1 CIDRβ3
var35	1	B	CD36	DBLα0.2 CIDRα3.1 DBLδ1 CIDRγ7
var37	1	B	CD36	DBLα0.3 CIDRα3.2 DBLδ1 CIDRγ5
var39	1	B	CD36	DBLα0.10 CIDRα2.8 DBLδ1 CIDRβ1
var41	1	C	CD36	DBLα0.16 CIDRα3.4 DBLβ10 DBLδ1 CIDRβ1
var42	1	A	EPCR	DBLα1.2 CIDRα1.7 DBLβ7 DBLγ2 DBLδ1 CIDRβ1
var44	1	B	CD36	DBLα0.23 CIDRα3.1 DBLδ1 CIDRβ5
var45	1	C	CD36	DBLα0.1 CIDRα3.1 DBLδ1 CIDRγ4
var51	0	C	CD36	DBLα0.1 CIDRα3.2 DBLδ6 CIDRβ2
		**HB3**		
var01	1	A	OTHER	DBLα1.6 CIDRδ2 DBLβ7 DBLγ13 DBLγ9 DBLδ1 CIDRβ
var08	1	B	CD36	DBLα0.9 CIDRα2.7 DBLδ1 CIDRγ7 DBLγ14 DBLζ5 DBLε4
var10	1	B	CD36	DBLα0.6 CIDRα3.1 DBLβ5 DBLδ1 CIDRβ6
var11	1	B	CD36	DBLα0.12 CIDRα2.1 DBLδ1 CIDRβ3 DBLε4
var16	1	B	CD36	DBLα0.9 CIDRα2.2 DBLδ1 CIDRβ1
var19	1	B	CD36	DBLα0.12 CIDRα2.2 DBLδ1 CIDRγ5
var22	0	B	CD36	DBLα0.8 CIDRα2.2 DBLδ3 CIDRγ2 DBLε2 DBLε7 DBLε3
var26	1	C	CD36	DBLα0.8 CIDRα3.5 DBLδ1 CIDRβ1
var27	1	B	CD36	DBLα0.1 CIDRα3.1 DBLδ1 CIDRβ1
var29	1	C	CD36	DBLα0.2 CIDRα3.2 DBLδ1 CIDRβ1
var30	1	B	CD36	DBLα0.1 CIDRα3.2 DBLδ1 CIDRβ1
var32	1	C	CD36	DBLα0.15 CIDRα3.1 DBLδ1 CIDRγ1

var33	1	C	CD36	DBLα0.22 CIDRα3.1 DBLδ1 CIDRγ6
var36	1	C	CD36	DBLα0.4 CIDRα3.2 DBLδ1 CIDRβ6
var40	1	B	CD36	DBLα0.7 CIDRα3.4 DBLδ1 CIDRγ1 DBLζ6 DBLε9
var50	1	B	CD36	DBLα0.1 CIDRα3.2 DBLδ1 CIDRβ6 DBLζ6 DBLε9
				**IGH**
var01	1	B	CD36	DBLα0.15 CIDRα3.2 DBLδ1 CIDRβ1
var03	1	B	CD36	DBLα0.5 CIDRα2.6 DBLδ1 CIDRβ1
var05	1	B	CD36	DBLα0.3 CIDRα3.4 DBLγ10 DBLδ1 CIDRγ5
var06	1	C	CD36	DBLα0.24 CIDRα3.1 DBLδ1 CIDRβ6
var08	1	B	CD36	DBLα0.14 CIDRα4 DBLδ1 CIDRβ6
var09	1	A	EPCR	DBLα1.2 CIDRα1.5 DBLβ6 DBLγ2 DBLγ4 DBLδ1 CIDRβ5
var16	1	C	CD36	DBLα0.11 CIDRα2.4 DBLδ1 CIDRβ6
var19	1	B	EPCR	DBLα2 CIDRα1.1 DBLβ1 DBLγ6 DBLδ1 CIDRβ1
var20	1	B	CD36	DBLα0.4 CIDRα3.1 DBLδ1 CIDRγ11
var23	1	A	EPCR	DBLα1.7 CIDRα1.4 DBLβ1 DBLγ11 DBLγ2 DBLδ1 CIDRβ1
var25	1	B	CD36	DBLα0.9 CIDRα2.2 DBLδ1 CIDRγ7
var27	1	A	EPCR	DBLα1.7 CIDRα1.4 DBLβ3 DBLβ6 DBLδ1 CIDRβ1
var36	1	C	CD36	DBLα0.22 CIDRα3.1 DBLδ1 CIDRγ7
var37	1	C	CD36	DBLα0.8 CIDRα5 DBLβ5 DBLγ5 DBLδ1 CIDRγ11
				**IT4/FCR3**
var05	1	C	CD36	DBLα0.5 CIDRα2.3 DBLδ1 CIDRβ1
var06	1	B	EPCR	DBLα2 CIDRα1.1 DBLβ12 DBLγ6 DBLδ1 CIDRβ4
var07	1	A	EPCR	DBLα1.7 CIDRα1.4 DBLβ1 DBLβ3 DBLγ10 DBLδ1 CIDRβ1
var12	1	ND	CD36	DBLα0.18 CIDRα6 DBLβ4 DBLδ1 CIDRβ1
var15	1	B	CD36	DBLα0.8 CIDRα3.5 DBLβ8 DBLδ1 CIDRβ1
var19	1	B	EPCR	DBLα2 CIDRα1.1 DBLβ12 DBLγ6 DBLδ1 CIDRβ1 DBLγ9
var21	1	ND	CD36	DBLα0.1 CIDRα3.1 DBLδ1 CIDRβ1
var24	1	B	CD36	DBLα0.10 CIDRα2.2 DBLδ1 CIDRγ5
var25	1	B	CD36	DBLα0.11 CIDRα2.4 DBLδ1 CIDRβ1
var26	1	B	CD36	DBLα0.23 CIDRα3.3 DBLδ1 CIDRβ1
var27	1	B	CD36	DBLα0.6 CIDRα3.1 DBLβ5 DBLγ5 DBLδ1 CIDRβ1
var29	1	B	CD36	DBLα0.3 CIDRα3.2 DBLδ1 CIDRγ5
var30	1	ND	CD36	DBLα0.13 CIDRα2.10 DBLδ1 CIDRβ1
var32a	1	ND	CD36	DBLα0.23 CIDRα3.2 DBLγ6 DBLδ1 CIDRβ1
var32b	1	B	EPCR	DBLα2 CIDRα1.1 DBLβ12 DBLγ6 DBLδ1 CIDRβ1
var33	1	B	CD36	DBLα0.11 CIDRα2.4 DBLδ1 CIDRβ5
var34	1	C	CD36	DBLα0.1 CIDRα3.1 DBLδ1 CIDRβ4
var39	1	ND	CD36	DBLα0.5 CIDRα2.5 DBLδ1 CIDRβ6
var40*	0	B	CD36	DBLα0.12 CIDRα2.11
var41	1	B	CD36	DBLα0.4 CIDRα5 DBLβ5 DBLγ5 DBLδ1 CIDRβ1
var44	1	B	CD36	DBLα0.16 CIDRα3.4 DBLβ13 DBLδ1 CIDRβ6
var45	1	B	CD36	DBLα0.5 CIDRα2.9 DBLδ1 CIDRβ1
var46	1	B	CD36	DBLα0.10 CIDRα2.2 DBLδ1 CIDRγ4 DBLε2 DBLζ3 DBLε3
var47	1	C	CD36	DBLα0.1 CIDRα3.3 DBLδ1 CIDRβ1
var51	1	C	CD36	DBLα0.17 CIDRα3.1 DBLδ1 CIDRγ12
var59	1	B	CD36	DBLα0.1 CIDRα3.1 DBLδ1 CIDRβ1
var62	1	B	CD36	DBLα0.1 CIDRα3.1 DBLδ8 CIDRβ2

				**3D7**
MAL6P1.1	1	B	CD36	DBLα0.8 CIDRα4 DBLδ1 CIDRβ1
MAL6P1.252	1	C	CD36	DBLα0.21 CIDRα2.1 DBLβ4 DBLδ1 CIDRβ1
MAL7P1.212	1	B	CD36	DBLα0.17 CIDRα3.1 DBLδ1 CIDRβ1
MAL7P1.55	1	B	CD36	DBLα0.9 CIDRα2.4 DBLδ1 CIDRγ5
MAL7P1.56	1	C	CD36	DBLα0.20 CIDRα3.1 DBLδ1 CIDRβ1
PF07_0048	1	C	CD36	DBLα0.1 CIDRα3.2 DBLδ1 CIDRβ6
PF07_49	1	C	CD36	DBLα0.17 CIDRα3.1 DBLδ1 CIDRγ7
PF07_51	1	C	CD36	DBLα0.1 CIDRα3.1 DBLδ1 CIDRβ1
PF08_0103	1	B	CD36	DBLα0.12 CIDRα2.2 DBLδ1 CIDRβ1
PF08_0142	1	B	CD36	DBLα0.9 CIDRα2.11 DBLδ1 CIDRβ1
PF10_0406	1	B	CD36	DBLα0.9 CIDRα2.7 DBLδ1 CIDRβ1
PF11_0007	1	B	CD36	DBLα0.15 CIDRα3.2 DBLδ1 CIDRβ1
PF11_0521	0	A	EPCR	DBLα1.7 CIDRα1.4 DBLβ3 DBLβ6 DBLδ3 CIDRγ2
PF13_0001	1	B	CD36	DBLα0.11 CIDRα2.4 DBLδ1 CIDRβ1
PF13_0003	0	A	OTHER	DBLα1.6 CIDRδ1 DBLβ3 DBLγ12 DBLδ5 CIDRβ3 DBLβ9
PFA0005w	1	B	CD36	DBLα0.11 CIDRα2.8 DBLδ1 CIDRβ1
PFB1055c	1	B	CD36	DBLα0.16 CIDRα3.4 DBLδ1 CIDRβ1
PFC0005w	1	B	CD36	DBLα0.9 CIDRα2.4 DBLδ1 CIDRβ1
PFD0020c	1	A	EPCR	DBLα1.2 CIDRα1.1 DBLβ12 DBLγ6 DBLγ11 DBLδ1 CIDRγ8
PFD615c	1	C	CD36	DBLα0.17 CIDRα3.1 DBLδ1 CIDRβ1
PFD630c	1	C	CD36	DBLα0.1 CIDRα3.1 DBLδ1 CIDRγ2
PFD635c	1	B	CD36	DBLα0.1 CIDRα3.1 DBLδ1 CIDRγ2
PFD995c	1	C	CD36	DBLα0.1 CIDRα3.2 DBLδ1 CIDRγ6
PFD1000c	1	C	CD36	DBLα0.1 CIDRα3.2 DBLδ1 CIDRγ11
PFD1005c	1	B	CD36	DBLα0.8 CIDRα4 DBLδ1 CIDRγ11
PFD1235w	1	A	EPCR	DBLα1.4 CIDRα1.6 DBLβ3 DBLβ3 DBLγ13 DBLδ1 CIDRβ5
PFE0005w	1	B	CD36	DBLα0.11 CIDRα2.4 DBLδ1 CIDRβ1
PFL0935c	1	B	CD36	DBLα0.16 CIDRα3.4 DBLδ1 CIDRγ12
PFL1955w	1	B	CD36	DBLα0.16 CIDRα3.4 DBLδ1 CIDRγ12
PFL1960w	1	C	CD36	DBLα0.20 CIDRα3.1 DBLδ1 CIDRβ1
PFL2665c	1	B	CD36	DBLα0.19 CIDRα2.3 DBLδ1 CIDRβ1
				**PFCLIN**
var26	1	C	CD36	DBLα0.16 CIDRα3.4 DBLδ1 CIDRγ5
var32	1	A	EPCR	DBLα1.2 CIDRα1.4 DBLβ7 DBLγ11 DBLδ1 CIDRβ1
var41	1	C	CD36	DBLα0.17 CIDRα3.1 DBLδ1 CIDRβ1
var44	1	B	CD36	DBLα0.5 CIDRα2.5 DBLβ13 DBLδ1 CIDRγ4
var47	1	C	CD36	DBLα0.17 CIDRα3.1 DBLδ1 CIDRβ1
var54	1	B	CD36	DBLα0.15 CIDRα3.2 DBLδ1 CIDRβ1
var55	1	C	CD36	DBLα0.5 CIDRα2.6 DBLδ1 CIDRβ1
var56	1	C	CD36	DBLα0.1 CIDRα3.1 DBLδ1 CIDRβ6
var59#	1	ND	OTHER	DBLβ5 DBLβ8 DBLδ1 CIDRβ2
var63	1	C	CD36	DBLα0.4 CIDRα3.1 DBLδ1 CIDRβ1
var64	1	C	CD36	DBLα0.19 CIDRα3.4 DBLδ1 CIDRγ6
var65	1	B	CD36	DBLα0.1 CIDRα3.1 DBLδ1 CIDRβ1
var66	1	C	CD36	DBLα0.5 CIDRα2.10 DBLβ13 DBLδ1 CIDRγ11
var77*	1	ND	?	DBLδ1 CIDRβ1

RAJ116				
var10	1	B	CD36	DBLα0.9 CIDRα2.1 DBLδ1 CIDRγ10
var11	1	B	EPCR	DBLα2 CIDRα1.8 DBLβ12 DBLγ4 DBLδ1 CIDRβ1
var13	1	B	CD36	DBLα0.1 CIDRα3.3 DBLδ1 CIDRβ6
var19	1	A	OTHER	DBLα1.6 CIDRδ1 DBLγ2 DBLδ1 CIDRβ1
var30	1	B	CD36	DBLα0.13 CIDRα2.6 DBLδ1 CIDRβ4 DBLε2
var31	1	B	CD36	DBLα0.5 CIDRα2.6 DBLδ1 CIDRβ5
var33	1	B	CD36	DBLα0.18 CIDRα6 DBLβ4 DBLγ13 DBLδ1 CIDRβ1
var34	1	B	CD36	DBLα0.15 CIDRα3.2 DBLδ1 CIDRγ10
var36	1	B	CD36	DBLα0.13 CIDRα2.9 DBLδ1 CIDRβ3
**Plasmodium reichenowi**				
var49	1	ND	CD36	CIDRα4 DBLδ1 CIDRβ5
var77	1	ND	?	DBLδ1 CIDRβ5
var85	1	ND	CD36	DBLα0.8 CIDRα5 DBLβ8 DBLδ1
var93	1	B	CD36	DBLα0.16 CIDRα3.4 DBLδ1 CIDRβ1

	PPRRRRLY	DBLδ1	UPS	Count	%	**P.falcip varA,B,C only**
Total	139	134	A	14	10.37037	**10.9375**
Pfalcip	135	130	B	76	56.2963	**59.375**
Preich	4	4	C	38	28.14815	**29.6875**
			ND	7	5.185185	
			Total	135		
			CD36	114	**85.07463**	
			EPCR	15	**11.19403**	
			OTHER	5	**3.731343**	
			Total	134		

* var genes that are only partially sequenced

PFCLINvar59 is predicted to be a DC8 EPCR binder but the distal n-terminal did not sequence well
